# The Role of Stress in Venipuncture Pain in Adolescents: Secondary Analysis of a Prospective Observational Study

**DOI:** 10.3390/children12060776

**Published:** 2025-06-14

**Authors:** Joel Brown, Zoe Ademuyiwa, Elizabeth Wu-Chen, Hadas Nahman-Averbuch

**Affiliations:** 1 Division of Clinical and Translational Research, Department of Anesthesiology, Washington University School of Medicine, St. Louis, MO 63110, USA; brownjoel@wustl.edu (J.B.); e.j.wu-chen@wustl.edu (E.W.-C.); 2 Washington University Pain Center, Department of Anesthesiology, Washington University School of Medicine, St. Louis, MO 63110, USA

**Keywords:** venipuncture pain, stress, blood draw, needle pain

## Abstract

**Background/Objectives:** Venipuncture is a painful and distress-inducing procedure, especially in adolescents. However, the effect of stress on venipuncture pain remains unclear. This study investigated the relationships between stress (venipuncture-related and general stress) and venipuncture pain intensity and unpleasantness, hypothesizing that higher stress levels would be associated with greater pain levels. **Methods**: Forty-two adolescents (five boys, mean age 12.2 ± 1.4) participated in the study, which included completing questionnaires and a blood draw. General stress was assessed using the Perceived Stress Scale. Before the blood draw, participants were asked to rate their venipuncture-related stress level using a Visual Analog Scale (VAS). Following venipuncture, participants rated their pain intensity and pain unpleasantness using the VAS. Nineteen participants returned for a similar study visit after 1 year. Regression models were used to assess the relationships between pain and stress. In addition, correlations were used to examine the relationships between baseline and 1-year follow-up stress and pain levels. **Results**: Only baseline venipuncture stress, but not general stress, was related to venipuncture pain intensity (estimate (SE) = 0.185 (0.046), t-ratio = 4.00, *p* < 0.001) and pain unpleasantness (estimate (SE) = 0.378 (0.116), t-ratio = 3.27, *p* = 0.002). Baseline stress levels were related to stress levels at 1-year follow-up. However, this was not found for pain levels. In addition, stress at baseline did not impact pain levels at 1-year follow-up. **Conclusions**: General stress may be different from venipuncture stress, with the latter having a greater influence on venipuncture pain. Developing interventions focused on reducing stress related to venipuncture in adolescents could assist in reducing pain and increase willingness to undergo needle procedures.

## 1. Introduction

Venipuncture can be a painful experience, which can lead to avoiding health-related procedures, such as vaccinations, and, ultimately, impact health, especially in the pediatric population [1,2,3,4]. Venipuncture is related to significant distress, which is defined as any type of negative effect related to the venipuncture procedure (e.g., fear, anxiety, stress) [5,6,7]. Anxiety is a behavioral state associated with the anticipation of potential, unspecified future threats, while fear is the response to a specific and actual threatening stimulus. Stress, on the other hand, is a broad term used to describe situations in which individuals feel that events in their lives are threatening or harmful and that they cannot cope effectively [8]. These fear and anxiety components related to venipuncture pain have been explored extensively [2,7,9,10,11,12,13,14]. However, the role of the stress component in venipuncture pain is less studied and could be critical in developing new interventions for venipuncture pain.

Higher levels of stress are related to greater clinical pain ratings in patients with endometriosis [15,16], visceral pain [17], back pain [18], and fibromyalgia [19]. In addition, stress is related to experimental pain, and higher levels of stress are associated with lower inhibitory pain modulation capabilities in healthy adults [20], as well as higher levels of pain ratings [21]. However, the relationships between stress levels and venipuncture pain are not clear.

Stress can be measured via the Perceived Stress Scale [22], which is not related to a specific event but instead assesses the general experience of chronic stress [8]. Another approach is individual judgment of specific events, in which the same event can be stressful for some individuals but not for others [8]. Previous studies have distinguished between specific and general psychological factors related to pain but found different effects of specific vs. general anxiety and pain catastrophizing levels on pain responses [23,24,25,26]. The present secondary analysis explored the relationships between stress, both general and specific, and venipuncture pain in healthy adolescents. In addition, the longitudinal relationships between stress and pain were also examined. We hypothesized that specific venipuncture-related stress would be related to venipuncture pain. In addition, we hypothesized that stress and pain levels at baseline would be related to stress and pain levels after 1 year.

## 2. Materials and Methods

This is a secondary analysis of a study aimed at identifying pubertal-related changes in pain [27,28]. The Institutional Review Board of Washington University in St. Louis approved the study. Before study participation, written informed consent and assent were obtained from all participants and their parents or guardians.

### 2.1. Participants

Participants were included in the study if they were healthy males or females between the ages of 9 and 16 with no diagnosis of chronic pain or frequent headaches (>5 headaches per month), no psychiatric or neurological disorders, no regular use of medications that affect nociceptive pathways (e.g., opioids, antidepressants), and no disorders associated with pubertal maturation.

### 2.2. Study Design

Participants completed a study visit at baseline and were invited to return after 1 year for an optional identical study visit. Each study visit included completing surveys, including the Perceived Stress Scale (PSS), the Pubertal Development Scale (PDS), the assessment of quantitative sensory testing (which will be presented elsewhere), and a blood draw, which always occurred at the end of the study visit.

#### 2.2.1. Perceived Stress Scale (PSS)

The PSS is a long-established self-report measure of perceived stress. This self-report questionnaire is used to assess the degree to which the respondent finds circumstances in their life to be unpredictable, uncontrollable, and/or overwhelming. The PSS captures a respondent’s subjective appraisal of whether life circumstances and experienced events exceed their adaptive capacity. Questions on the PSS ask the respondent to rate their feelings and thoughts for the past month, indicating that general stress (i.e., not related to a specific event) for a relatively recent time frame is assessed [2]. Individual scores on the PSS can range from 0 to 40, with higher scores indicating higher perceived stress. Scores ranging from 0 to 13 are considered low stress, 14 to 26 are considered moderate stress, and 27 to 40 are considered high stress. The PSS has been used in adolescent studies and is validated in this population [29,30,31].

#### 2.2.2. Pubertal Development Scale (PDS)

Pubertal maturation stage was assessed using the Pubertal Development Scale (PDS) questionnaire [32]. This survey has good reliability and validity and is correlated with Tanner staging [32,33]. Higher scores indicate a greater pubertal maturation stage.

#### 2.2.3. Venipuncture

At the end of the study visit, a blood draw was completed. The blood draw was conducted in the presence of the parent or guardian in the study room. A trained nurse (J.B.) performed all blood draws. After the nurse explained the venipuncture procedure, participants were asked to rate their specific stress level for the blood draw (termed “specific stress”) using a Visual Analog Scale (VAS) ranging from “no stress at all” to “the most stress imaginable,” which is similar to previous studies [31,34]. Then, the nurse tied a tourniquet around the participant’s arm and located an adequate vein for venipuncture. After locating an adequate vein and removing the tourniquet, the participants were asked if they would like numbing cream and/or spray. The spray used was McKesson Topical Anesthetic spray. The cream used was Nivagen Lidocaine 5% topical anesthetic cream. While taking time for the cream or spray to take effect, participants were given snacks and/or drinks. Venipuncture was performed using a 21- or 23-gauge BD Vacutainer Push Button blood collection set. Following venipuncture, participants were asked to rate their pain intensity and pain unpleasantness levels from venipuncture using the VAS, ranging from “no pain intensity/no pain unpleasantness” to “most intense pain sensation imaginable/most unpleasant sensation imaginable”.

#### 2.2.4. Pain Rating Scales

Venipuncture pain intensity and pain unpleasantness ratings were collected separately using a mechanical VAS. Participants were instructed to slide the inner portion of the VAS from no pain/unpleasantness to the most intense pain/unpleasantness imaginable. As the participant slides the scale to the right, it exposes more color (on the participant-facing side), which corresponds to greater pain intensity/unpleasantness. Ratings corresponded to numeric values 0–10, accurate to one decimal place (on the experimenter-facing side, and participants did not see these values). The VAS has ratio scale properties, high reliability, and validity for detecting changes in pain magnitude [35,36,37,38,39,40,41].

### 2.3. Statistical Analysis

Data analysis was completed using JMP (Version 16.0.0). Univariate correlations were conducted to identify factors that are related to the study outcomes (venipuncture pain intensity and unpleasantness ratings). Venipuncture pain intensity and unpleasantness ratings were not related to age and sex (Supplementary Table S1); thus, they were not included in the analyses. For the analgesic agent, 35 participants used both cream and spray as an analgesic numbing agent, 2 participants used only cream, and 5 participants deferred to no analgesic agent. Venipuncture pain intensity ratings were related to analgesic agents, and thus the numbing agent was controlled for in the venipuncture pain intensity analysis. To identify the role of stress in venipuncture pain, regression models were conducted using venipuncture pain intensity or pain unpleasantness ratings as the dependent variables and specific stress (related to venipuncture) and general stress (using the PSS) as the independent variables. In order to verify that the numbing agent did not impact the results, we stratified the participants based on the numbing agent that was used and repeated the regression models, including only participants who used both the cream and spray (n = 35). Conducting the models for the other numbing agent options was not feasible due to the small n (n = 2 for only cream and n = 5 for no numbing agent).

T-tests were used to compare stress and pain levels at baseline and 1-year follow-up. In addition, they were used to compare stress and pain scores at baseline between participants who returned to complete the 1-year follow-up and participants who did not. To identify the factors impacting venipuncture pain at the 1-year follow-up, we tested correlations between year 1 venipuncture pain and (1) stress specific to venipuncture at baseline, (2) stress specific to venipuncture at 1-year follow-up, and (3) venipuncture pain levels at baseline. At the 1-year follow-up, pain intensity and unpleasantness levels were not related to sex, age, or analgesic agent used (Supplementary Table S1), and thus they were not controlled for in the models.

## 3. Results

Forty-two adolescents completed the baseline study visit (five boys, mean age 12.2 ± 1.4). All participants were non-Hispanic, 29 were white, 8 were black, 4 were Asian/Pacific Islander, and 1 was mixed. Furthermore, 11 participants had an annual household income of < USD 100,000, 10 had an income between USD 100,000 and USD 149,000, and 19 had > USD 150,000 (n = 2 refused to answer). The baseline mean PSS was 19.2 ± 4.6, which indicates moderate stress. The mean venipuncture stress before the blood draw was 3.0 ± 3.0. The mean venipuncture pain intensity was 0.8 ± 0.9, and the mean pain unpleasantness was 1.6 ± 2.3. No relationships were found between PDS scores and baseline pain intensity (r^2^ = 0.018, *p* = 0.393), pain unpleasantness (r^2^ = 0.001, *p* = 0.850), specific stress (r^2^ = 0.010, *p* = 0.543), or general stress (r^2^ = 0.009, *p* = 0.540).

### 3.1. Relationships Between Stress and Pain at Baseline

At baseline, general stress was not related to baseline venipuncture stress (r^2^ = 0.065, *p* = 0.111). Thus, both were included in the regression models as independent variables explaining pain levels. Both baseline pain intensity and unpleasantness ratings were related only to baseline venipuncture stress and not baseline general stress (Table 1). Even though the numbing agent did not impact the results, we aimed to stratify the results based on the agent. Due to the low number of participants who chose to use only cream (n = 2) and no analgesic agent (n = 5), the analyses were rerun, including only participants who used both cream and spray (n = 35). Similar results were found with both baseline pain intensity and unpleasantness ratings, and they were related only to baseline venipuncture stress and not baseline general stress (Supplementary Table S2). Furthermore, including PDS scores in the models did not change the results, and baseline venipuncture stress was still significantly related to venipuncture pain intensity and unpleasantness ratings.

### 3.2. Relationships Between Stress and Pain at Baseline and 1-Year Follow-Up

**Figure 1:**
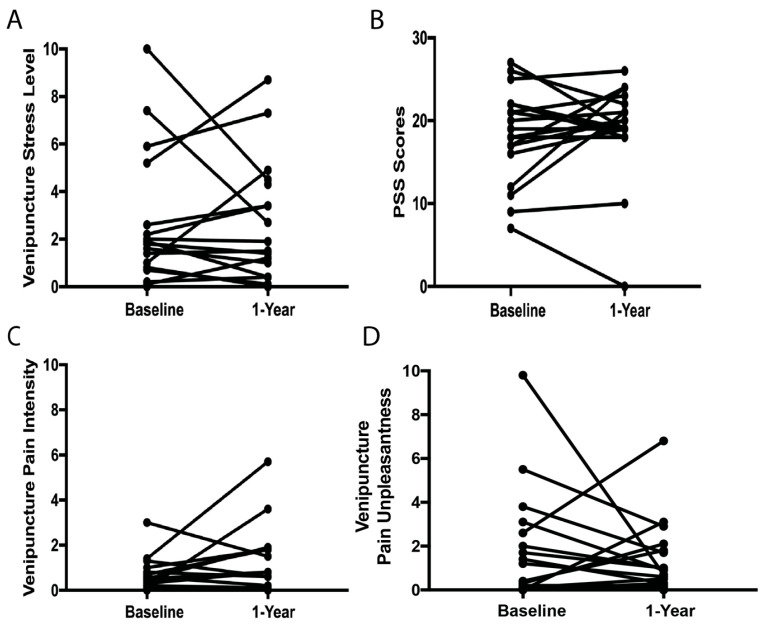
**Changes in stress and pain levels from baseline to 1-year follow-up.** (**A**) Venipuncture stress (0–10 Visual Analog Scale). (**B**) General stress assessed via the Perceived Stress Scale (PSS). (**C**) Venipuncture pain intensity (0–10 Visual Analog Scale). (**D**) Venipuncture pain unpleasantness (0–10 Visual Analog Scale).

**Figure 2:**
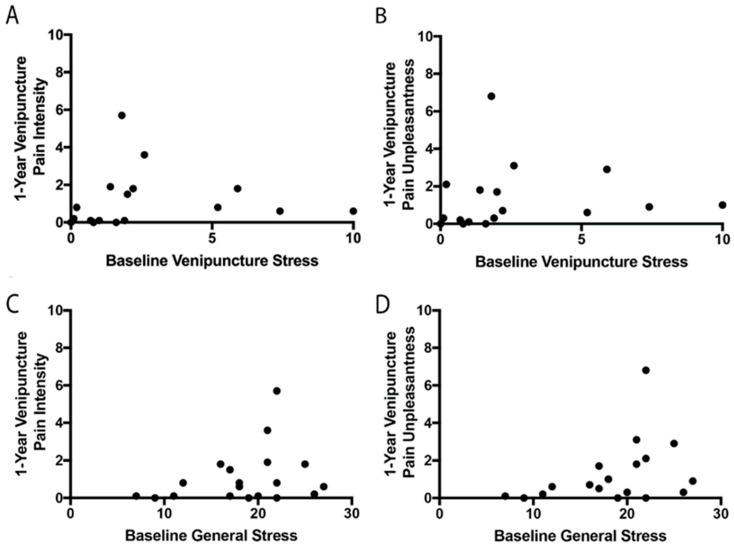
No significant associations between stress at baseline and venipuncture pain after 1 year. (**A**) Association between venipuncture stress at baseline and venipuncture pain intensity after 1 year. (**B**) Association between venipuncture stress at baseline and venipuncture pain unpleasantness after 1 year. (**C**) Association between general stress at baseline and venipuncture pain intensity after 1 year. (**D**) Association between general stress at baseline and venipuncture pain unpleasantness after 1 year.

Nineteen participants returned for the additional identical study visit at 1-year follow-up (four boys). No differences in baseline stress and pain scores were found between participants who completed and did not complete the 1-year follow-up (Supplemental Table S3). The stress and pain scores at baseline and after 1 year in these participants are summarized in Supplemental Table S4 and Figure 1. Even though some participants had an increase in their pain and stress levels from baseline to 1-year follow-up, others had a decrease. No significant differences were found between pain and stress levels at baseline and 1-year follow-up. Specific venipuncture stress ratings at baseline were related to specific venipuncture stress ratings at the 1-year follow-up (r^2^= 0.389, *p* = 0.007, Table 2). Similarly, higher levels of general stress at baseline were related to higher levels of general stress at year 1 follow-up (r^2^= 0.322, *p* = 0.011, Table 2). Surprisingly, venipuncture pain intensity and unpleasantness ratings at baseline were not correlated with venipuncture pain intensity ratings at year 1 follow-up (Table 2, Figure 2). In addition, baseline stress levels, both specific and general stress, were not related to venipuncture pain intensity or unpleasantness ratings at year 1.

## 4. Discussion

This study examines the relationships between stress and venipuncture pain. Using both general and specific stress levels, it was found that only specific venipuncture stress, but not general stress, was related to venipuncture pain at baseline. Interestingly, stress levels at baseline were related to stress levels at 1-year follow-up, but this was not found for pain levels.

Stress describes a situation in which an individual feels that events in their lives are harmful and that they cannot cope with them effectively. Both general and specific stress are related to increased risk for morbidity and mortality, as well as disease prognosis [42,43,44,45]. Pain, specifically needle pain, can be considered a stressor that can evoke a stress response or distress [9,12,46,47,48]. In addition, greater pain levels are related to higher stress levels in patients with chronic pain and healthy controls [15,16,17,18,19,20,21]. In the present study, we focused on two aspects of stress, venipuncture-specific and general. Interestingly, these two types of stress were not correlated, and only venipuncture stress levels, not general stress, were related to venipuncture pain levels. Thus, assessing the specific stress levels in response to a specific stressor, such as venipuncture, could be more relevant and have a greater impact on the individual experience of venipuncture pain than general stress. Similar results were found for pain catastrophizing in studies that distinguished between situational pain catastrophizing, which is specific to noxious stimulation, and dispositional catastrophizing, which refers to catastrophizing thoughts during previous pain events. Situational pain catastrophizing was not related to dispositional pain catastrophizing, and experimental pain sensitivity was more strongly associated with situational pain catastrophizing than dispositional pain catastrophizing [23,24,25]. Thus, psychological factors related to a specific situation may have a different impact on pain compared to general psychological factors.

Many individuals experience distress before venipuncture, which includes behaviors of fear, anxiety, and stress. Our results show that higher stress levels are related to higher venipuncture pain. Similar results were found for fear and anxiety, which were related to venipuncture pain and rated higher [10,49], suggesting that venipuncture can evoke a strong behavioral response involving stress, anxiety, and fear. In line with this, interventions, such as distraction, cognitive behavioral therapy, and pharmacological interventions, aim to reduce venipuncture pain and distress, which includes anxiety, fear, and stress [9,50,51,52,53]. The results of this study suggest that interventions should target specific situational venipuncture-related distress rather than general distress.

In a sub-sample that returned for the optional follow-up study visit after 1 year, there were no differences between stress and pain levels at baseline and 1-year follow-up. Notably, large individual variability in the changes was found, with some participants demonstrating an increase while others demonstrated a decrease in pain or stress levels. Despite this large variability, the stress levels at baseline were related to the stress levels after 1 year, indicating a stable response to stress, as was also found in previous longitudinal short- and long-term studies [44,54]. On the contrary, venipuncture pain ratings at baseline were not related to pain or stress ratings after 1 year, which could be due to age/developmental changes, recall bias, or placebo effect. During pubertal maturation or aging, there are major changes in biopsychosocial factors that could impact pain processing and pain sensitivity [55]. Interestingly, previous cross-sectional studies of adolescents scheduled to undergo venipuncture for routine care found an age effect on venipuncture pain such that older adolescents had lower venipuncture pain intensity compared to younger adolescents or children [13,14], which is in line with the reported age effects on experimental pain sensitivity [55,56,57]. Another factor that could affect pain and stress levels at the 1-year follow-up study visit is the memory of the painful experience during the baseline study visit. Previous studies have found that children and adolescents can recall their pain ratings to experimental pain procedures after 1 year [58]. For medical procedures, such as venipuncture, the memory of past pain experiences can predict future pain levels [59]. For example, in children, if, during baseline, pain was not adequately managed, higher pain ratings were found at follow-up visits [60]. In addition, individuals can differ in their placebo response. Although pediatric participants have, overall, a higher placebo response compared to adults [61], there is also individual variability in the placebo response within each group. A placebo response to a pharmacological intervention for needle pain was found in children and adolescents, and there was no difference found in venipuncture pain between lidocaine and placebo administration [62]. Thus, pain, compared to stress, may be more impacted by various factors that exhibit temporal longitudinal changes in adolescents.

It is important to note that the venipuncture procedure in the present study was optional and for research purposes only, and an analgesic agent was offered to increase participants’ recruitment and retention. The use of analgesic agents could also explain the low venipuncture pain and stress levels observed at both baseline and 1-year follow-up. However, even with an analgesic agent, variability in both venipuncture stress and pain was observed. Even without using analgesic agents, which would result in higher pain ratings, we expect similar relationships to exist in which specific stress is the only factor related to venipuncture pain, suggesting that stress may have a greater impact on pain even if an analgesic agent is used.

The main limitations of the present study are the small sample size and the exploratory approach, which need to be replicated in future studies. This study is a secondary analysis of a study aimed at identifying pubertal and hormonal effects on pain sensitivity in adolescents. Thus, a power calculation was not conducted for the aims explored in the present study, and even though significant relationships were identified, the study may be underpowered. In addition, the number of blood draws the study participants experienced before participating in the present study or between the baseline and the 1-year follow-up study visits was not assessed. It is possible that participants who experience more blood draws will develop coping strategies and have lower stress and pain from venipuncture procedures. However, as the study participants were healthy, we anticipate that most participants will have had no or only routine blood draws, which should be similar across all participants. Importantly, the present research study may have a selection bias as participants with severe needle fear or anxiety most likely chose not to participate in this study, although the presence or lack of needle fear or anxiety was not confirmed.

## 5. Conclusions

Specific venipuncture-related stress is different from general stress and is the only factor related to venipuncture pain. Developing interventions focused on reducing stress-related venipuncture in adolescents rather than general stress could assist in reducing venipuncture pain and increase willingness to undergo needle procedures.

## Figures and Tables

**Table 1:** Regression models of the relationships between baseline stress and pain.

	Estimate	SE	T Ratio	*p* Value
**Baseline Venipuncture Pain Intensity (n = 40)**				
Specific stress	0.185	0.046	4.00	**<0.001**
General stress	0.005	0.029	0.17	0.864
Agent [both]	−0.234	0.238	−0.98	0.332
Agent [cream]	0.598	0.394	1.52	0.138
**Baseline Venipuncture Pain Unpleasantness (n = 40)**				
Specific stress	0.378	0.116	3.27	**0.002**
General stress	−0.016	0.074	−0.21	0.831

Specific stress: stress levels (0–10 VAS) before venipuncture; general stress: Perceived Stress Scale score. Including PDS scores did not change the results. Bold indicates statistical significance.

**Table 2:** Correlations between stress and pain levels at baseline and after 1 year.

	Year 1 General Stress	Year 1 Specific Stress	Year 1 Venipuncture Pain Intensity	Year 1 Venipuncture Pain Unpleasantness
**Baseline general stress (n = 19)**	**R^2^ = 0.322, *p* = 0.011**	R^2^ = 0.026, *p* = 0.508	R^2^ = 0.082, *p* = 0.234	R^2^ = 0.142, *p* = 0.112
**Baseline specific stress (n = 17)**	R^2^ = 0.076, *p* = 0.284	**R^2^ = 0.389, *p* = 0.007**	R^2^ = 0.002, *p* = 0.853	R^2^ = 0.007, *p* = 0.747
**Baseline venipuncture pain intensity (n = 19)**	R^2^ = 0.025, *p* = 0.517	R^2^ = 0.005, *p* = 0.763	R^2^ = 0.133, *p* = 0.124	R^2^ = 0.133, *p* = 0.125
**Baseline venipuncture pain unpleasantness (n = 19)**	R^2^ = 0.043, *p* = 0.397	R^2^ = 0.095, *p* = 0.200	R^2^ = 0.092, *p* = 0.206	R^2^ = 0.043, *p* = 0.395

## Data Availability

Data will be shared upon reasonable request to the investigators due to privacy.

## Supplementary Materials

The following supporting information can be downloaded at https://www.mdpi.com/article/10.3390/children12060776/s1, Table S1: Relationships between pain and confounding variables; Table S2: Relationships between baseline stress and pain scores in adolescents using both cream and spray agent; Table S3: Baseline stress and pain levels in adolescents completing vs. not completing the 1-year follow-up visit; Table S4: Stress and pain levels at baseline and Year 1.

## Abbreviations

The following abbreviations are used in this manuscript:

VAS	Visual Analog Scale
PSS	Perceived Stress Scale
PDS	Pubertal Development Scale
