# Supplementary material for: The Role of Stress in Venipuncture Pain in Adolescents: Secondary Analysis of a Prospective Observational Study
Source: Children (Basel). 2025 Jun 14;12(6):776. doi: 10.3390/children12060776 (PMC12191137; doi:10.3390/children12060776)
Supplement: Supplementary file 1 [file children-12-00776-s001.zip › children-3687467-supplementary.pdf]

Supplementary Table S1

	Estimate	SE	T ratio	P value
**Baseline Venipuncture Pain intensity**				
Age	-0.074	0.110	-0.68	0.503
Sex	-0.247	0.219	-1.13	0.267
Agent [Both]	-0.318	0.283	-1.12	0.268
Agent [Cream]	1.181	0.452	2.61	**0.013**
**Baseline Venipuncture Pain unpleasantness**				
Age	0.184	0.275	0.67	0.508
Sex	-0.760	0.548	-1.39	0.174
Agent [Both]	0.154	0.708	0.22	0.829
Agent [Cream]	1.363	1.131	1.21	0.236
**Year 1 Venipuncture Pain intensity**				
Age	-0.058	0.291	-0.20	0.846
Sex	-0.250	0.502	-0.50	0.627
Agent	-0.385	0.555	-0.69	0.501
**1 year Venipuncture Pain unpleasantness**				
Age	0.037	0.324	0.11	0.911
Sex	-0.387	0.560	-0.69	0.501
Agent	-0.244	0.619	-0.39	0.700

At baseline, 35 participants used both cream and spray as an analgesic numbing agent, 2 participants used only cream, and 5 participants deferred to no analgesic agent. At 1 year follow-up, 3 participants used both cream and spray as an analgesic numbing agent and 14 participants used only cream.

Supplementary Table S2. Relationships between baseline stress and pain scores in adolescents using both cream and spray agent

	Estimate	SE	T ratio	P value
**Baseline Venipuncture Pain intensity (n=35)**				
Specific stress	0.178	0.052	3.39	**0.002**
General stress	0.001	0.031	0.03	0.980
**Baseline Venipuncture Pain unpleasantness (n=35)**				
Specific stress	0.376	0.151	2.49	**0.019**
General stress	-0.020	0.090	-0.22	0.829

Specific stress- stress levels (0-10 VAS) before venipuncture; General stress- perceived stress scale score

Supplementary Table S3. Baseline stress and pain levels in adolescents completing vs. not completing the 1-year follow-up visit

	Completing 1-year study visit (n=19)	Not completing 1-year study visit (n=23)	P value
Baseline general stress	18.4±5.6	19.9±3.6	0.320
Baseline specific stress	2.6± 2.8	3.3±3.2	0.500
Baseline venipuncture pain intensity	0.6±0.7	1.0 ±1.1	0.157
Baseline venipuncture pain unpleasantness	1.7±2.5	1.5±2.1	0.777

Data is presented as mean± SD. Specific stress- stress levels (0-10 VAS) before venipuncture; General stress- perceived stress scale score

Supplementary Table S4. Stress and pain levels at baseline and Year 1

	Baseline (mean± SD)	Year 1 (mean± SD)	P value
General stress	18.4±5.6	18.9±5.7	0.666
Specific stress	2.6± 2.8	2.5±2.5	0.839
Venipuncture pain intensity	0.6±0.7	1.1 ±1.5	0.149
Venipuncture pain unpleasantness	1.7±2.5	1.3±1.6	0.495

Specific stress- stress levels (0-10 VAS) before venipuncture; General stress- perceived stress scale score
